# Sex-Related Differences Between Patients With Symptomatic Acute Aortic Dissection

**DOI:** 10.1097/MD.0000000000003100

**Published:** 2016-03-18

**Authors:** Buamina Maitusong, Hui-Ping Sun, Dilidaer Xielifu, Maisumu Mahemuti, Xiang Ma, Fen Liu, Xiang Xie, Adila Azhati, Xin-Rong Zhou, Yi-Tong Ma

**Affiliations:** From the Department of Cardiology, First Affiliated Hospital of Xinjiang Medical University, Urumqi 830054, People's Republic of China (BM, Y-TM, MM, H-PS, XM, XX, AA, X-RZ, DX); Xinjiang Key Laboratory of Cardiovascular Disease Research, Urumqi 830054, People's Republic of China (Y-TM, FL).

## Abstract

We designed a retrospective cohort study to assess sex-related differences in clinical manifestations, incidence, and outcomes of patients with symptomatic acute aortic dissection (AAD).

We collected clinical data from 2010 to 2015 of 400 patients with AAD. Patients’ clinical characteristics, treatment, and outcomes were analyzed as a function of sex.

Among 400 patients with AAD, the ratio of men to women was 3.18:1; the incidence of atherosclerosis was higher in women (*P* = 0.02). Dysphoria (*P* = 0.01), focal neurological deficits (*P* = 0.04), and pulse deficits (*P* = 0.03) were more frequent in men. Imaging findings revealed that pleural effusion (*P* < 0.01), celiac trunk involvement (*P* < 0.01), and superior mesenteric artery involvement (*P* = 0.02) were more frequent in men. Dissection-related pneumonia (*P* = 0.02), pulmonary atelectasis (*P* = 0.01), aortic intramural hematoma (*P* < 0.01), ischemic electrocardiographic changes (*P* = 0.03), and in-hospital complications such as myocardial ischemia (*P* = 0.03), hypoxemia (*P* < 0.01), cardiac tamponade (*P* = 0.01) occurred more frequently in women. Women with type A dissection had higher in-hospital mortality than men (*P* < 0.01).

The presentation of AAD varies with a patient's sex. Women with AAD had clinical features different from men as follows: higher age of onset, more frequent inpatient complications, and higher in-hospital mortality. These findings may lead to a better understanding of aortic dissection in women that will improve their outcomes.

## INTRODUCTION

Acute aortic dissection (AAD) is a life-threatening condition associated with morbidity and mortality. AAD was considered a rare disease (2.9 to 3.5 cases per 100,000 person-years),^1^ However, according to the United States Centers for Disease Control and Prevention, diseases of the aorta and its branches account for 43,000 to 47,000 deaths annually in the United States,^2^ indicating that AAD is not “rare.” Most studies of autopsies suggest that the presentation of aortic disease is often death because of AD and rupture.^3,4^ The overall prognosis of patients with AAD is poor, and ∼40% of patients will die immediately, 1% per hour will die after onset, and 5% and 20% die during or shortly after surgery, respectively.^5^

The first cases of aortic dissection was reported in the 18th century,^6^ with the development of the diagnosis and therapeutic method, interest and research in aortic dissection increased gradually.^7–9^ These advancements facilitate early diagnosis, exquisite treatment, and improved prognosis.^6,10^ Regardless of mounting research in AAD, still insufficient information is available to determine if there are sex-related differences in clinical manifestations, and prognosis.^6^ Accordingly, the aim of the present study was to assess differences between male and female patients with AAD in Xin Jiang, China. We expected that our study would provide valuable information regarding sex-related differences in Symptomatic Acute Aortic Dissection.

## METHODS

### Ethical Approval of the Study Protocol

This study was approved by the Ethics Committee of the First Affiliated Hospital of Xinjiang Medical University (Urumqi, China) and was conducted according to the standards of the Declaration of Helsinki.

### Study Population and Data Collection

We collected clinical data on all patients with acute aortic dissection diagnosed and treated at the First Affiliated Hospital of Xinjiang Medical University from 2010 to 2015 (n = 400). This hospital is the first diagnostic and treatment center for cardiovascular disease in the Xinjiang Uygur Autonomous Region of China and is staffed by a highly experienced and successful medical team that provides advanced medical facilities to patients from every region of Xin Jiang (23 cities, 7 regions, 5 autonomous prefectures, and 68 counties). Data were collected using standardized forms that included information about patient demographics, history, clinical presentation, imaging findings, management, clinical events, and mortality. Patients were identified by searching hospital discharge diagnostic record, surgical and echocardiography laboratory databases. Diagnosis was determined using history, findings of imaging studies, and observations during surgery.

### Inclusion and Exclusion Criteria

Inclusion criteria were any symptomatic acute aortic dissection (2 weeks from onset of symptoms, including Stanford types A and B). Exclusion criteria were any chronic dissection (duration >2 weeks), any asymptomatic patients, or aortic disruption secondary to trauma.

### Anatomical Classification of Aortic Dissection

AAD is classified according to the origin of the intimal tear or whether the dissection involves the ascending aorta (regardless of site of origin). Accurate classification is important, because it influences decisions regarding surgical versus nonsurgical management. In the present study, we used the Stanford classification system that categorizes dissections as those that do or do not involve the ascending aorta as follows: Type A, all dissections involving the ascending aorta regardless of the site of origin; and Type B, all dissections that do not involve the ascending aorta. (Involvement of the aortic arch without involvement of the ascending aorta is designated Type B.)^11^.

### Statistical Analysis

The summary of the statistical analyses includes the values of the mean and standard deviation (SD) of frequencies and percentages. Missing data were not defaulted to negative, and denominators reflect only reported cases. Univariate associations between the 2 groups for nominal variables were compared using the Pearson χ^2^ test or Fisher's exact test, and the 2-tailed Student *t* test was used to evaluate continuous variables. Data analysis was performed using Statistical Package for Social Sciences (SPSS) v. 17.0 for Windows (SPSS Institute, Chicago). *P* < 0.05 was considered statistically significant.

## RESULTS

### Sex-Specific Differences Among Age Groups

**FIGURE 1:**
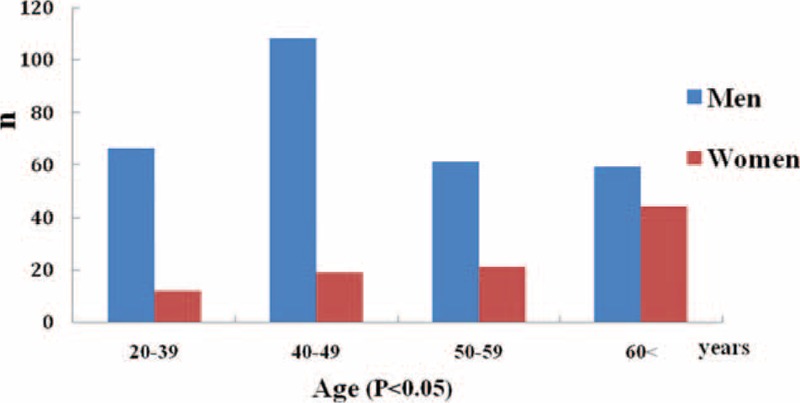
Differences in age of onset between women and men. Total 400 patients, ages ranged from 22 to 91 years, the proportion of men was significantly higher in the 20 to 39, 40 to 49 age groups compared with that of women (*P* < 0.05), and the proportion of women with AAD was slightly increased after 60 years of age. AAD = acute aortic dissection.

As shown in Figure 1, of 400 patients with ADD, ages ranged from 22 to 91 years. The incidence of AAD was significantly higher in men aged 20 to 39, 40 to 49 years compared with that of women (*P* < 0.05), the proportion of women with AAD was slightly increased after 60 years of age. As shown in Table 1, there were significant differences in the average age of onset between the men and women (men 49.6 ± 12.6 years; women 54.2 ± 12.4 years, *P* < 0.01).

**TABLE 1:**
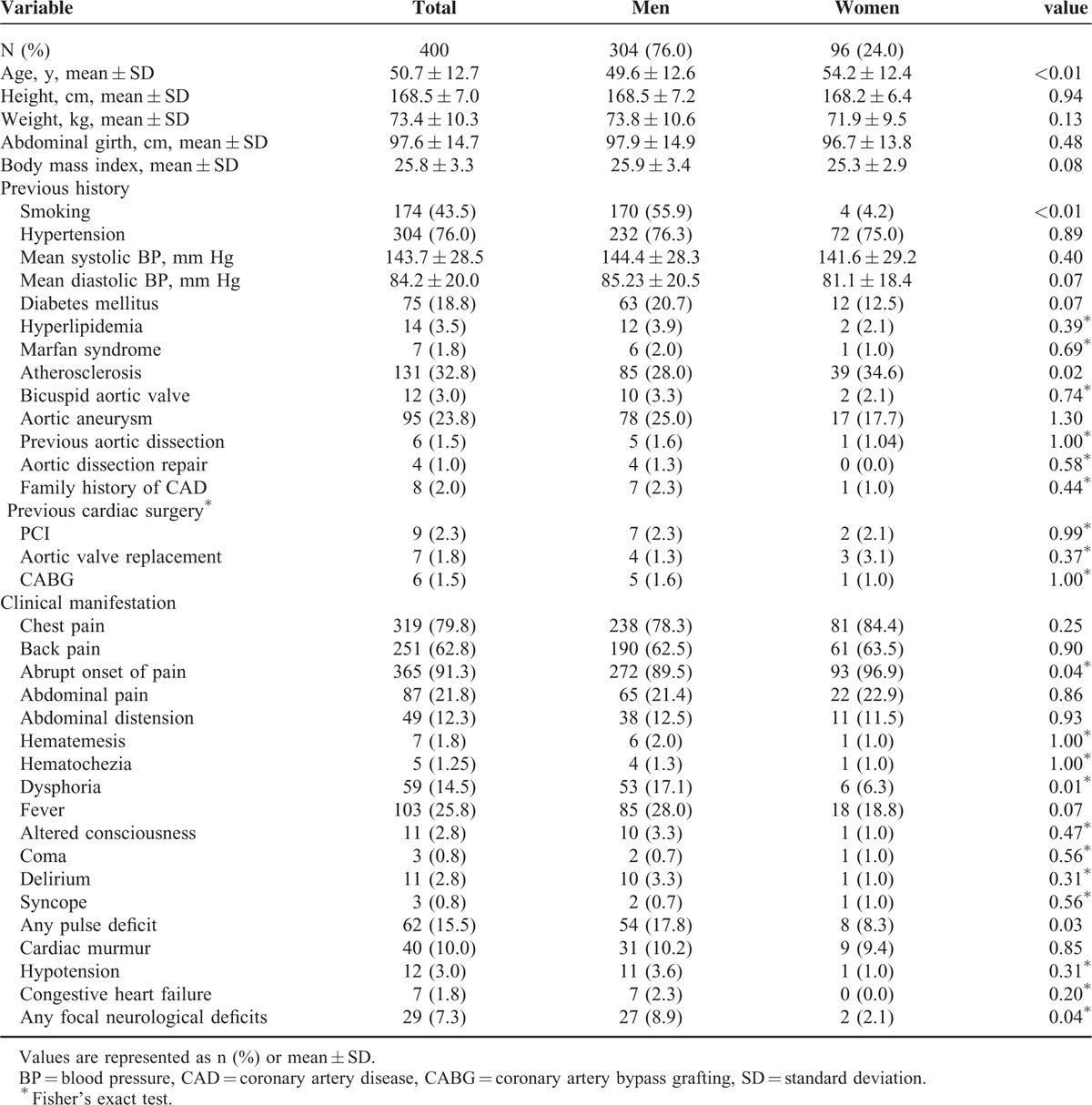
Patients’ Histories and Clinical Manifestations Between Women and Men

### Sex-Related Differences in Patients’ Histories and Clinical Characteristics

As shown in Table 1, of 400 patients with ADD, 76% were men and 24% were women, and the ratio of men to women was ∼3.18:1. According to their previous medical histories, the percentage of men who smoked was significantly higher than that of women (55.9% vs 4.17%, *P* < 0.01), and the percentage of women with arthrosclerosis^12^ was higher than that of men (40.6% vs 28.21%, *P* = 0.02). There were no significant differences in other variables, including Marfan syndrome,^13–14^ bicuspid aortic valve,^15–16^ aortic aneurysm,^17–18^ and cardiac surgeries (percutaneous coronary intervention, coronary artery bypass grafting, and aortic valve replacement, etc).

The mean values of systolic and diastolic blood pressures upon presentation did not differ between groups, whereas the classic presentation of chest or back pain at onset was similar, although women experienced abrupt onset of pain more frequently than men (96.9% vs 89.5%, *P* = 0.04). Dysphoria (17.1% vs 6.3%, *P* = 0.01), focal neurological deficits (8.9% vs 2.1%, *P* = 0.04), and pulse deficit symptoms (17.8% vs 8.3%, *P* = 0.03) were more frequent in men. There were no significant differences in the frequencies of uncommon symptoms upon onset such as abdominal pain, hematochezia, coma, and congestive heart failure.

### Sex-Related Differences in Diagnostic Imaging and Electrocardiographic Data

As shown in Table 2, diagnostic imaging studies were used similarly, and the findings suggested that pleural effusion (19.1% vs 7.3%, *P* < 0.01), celiac trunk involvement (33.9% vs 20.8%, *P* = 0.02), and superior mesenteric artery involvement (27.3% vs 15.6%, *P* = 0.02) were more frequent in men. In contrast, widened aortic shadow (49.6% vs 64.6%, *P* = 0.01), comorbid pneumonia (18.2% vs 29.2%, *P* = 0.02), pulmonary atelectasis (49.2% vs 64.6%, *P* = 0.01), compromised coronary artery (3.9% vs 10.4%, *P* = 0.02), intramural hematoma (10.5% vs 21.9%, *P* < 0.01), and pericardial effusion (20.2% vs 33%, *P* < 0.01) were more frequent in women. The ECG findings showed new Q waves or elevation of the ST-segment (4.6% vs 10.4%, *P* = 0.04), low voltage (2.0% vs 9.4%, *P* < 0.01), and ischemic changes (6.6% vs 15.6%, *P* < 0.01) were more frequent among women (Table 2).

**TABLE 2:**
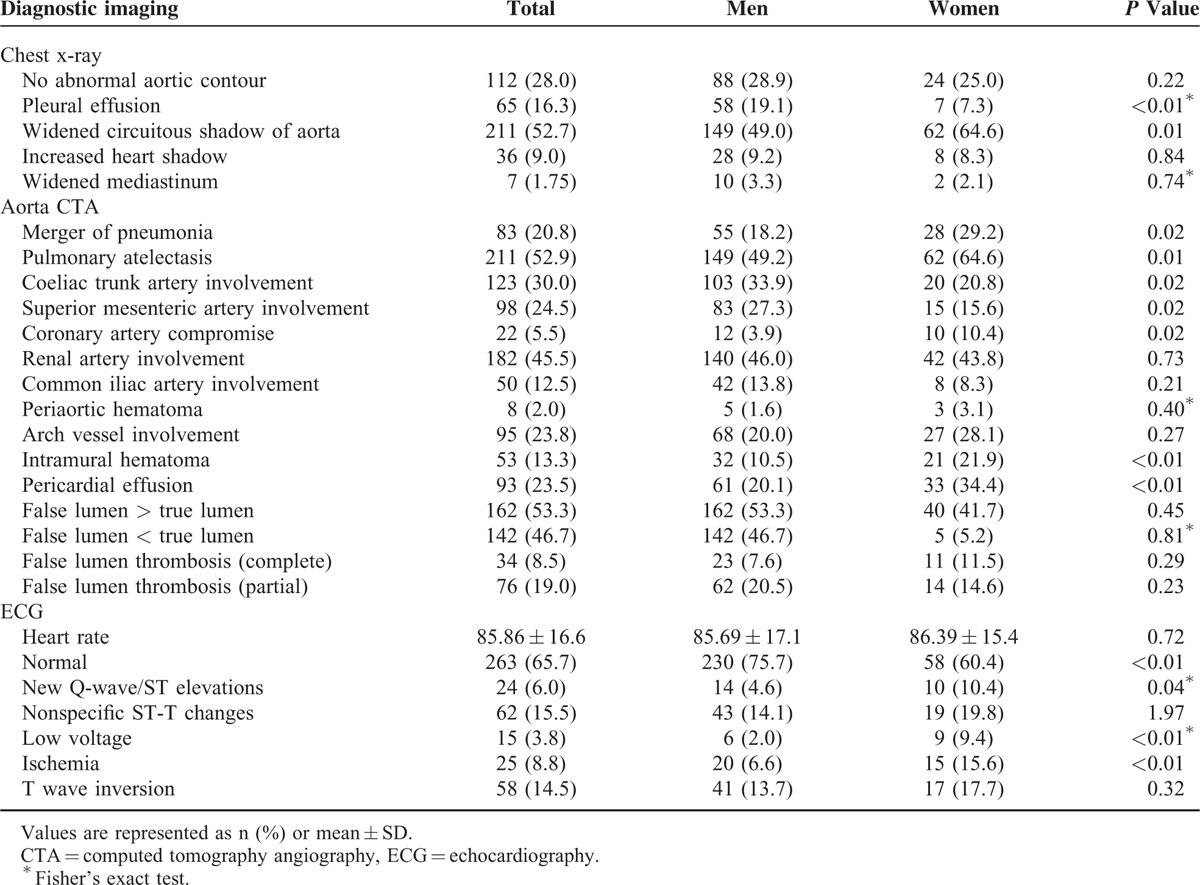
Imaging Findings for Men and Women

### Sex-Related Differences Among in-Hospital Complications and Mortality

There were no significant differences in the classifications of dissection, hours from onset symptoms to diagnosis. The frequencies of in-hospital treatments such as surgery and endovascular treatment were not significantly different between men and women (Table 3). Severe in-hospital complications such as myocardial ischemia/infarction, hypoxemia, and cardiac tamponade occurred more frequently among women (all *P* < 0.05). In contrast, men experienced mesenteric ischemia/infarction and acute renal damage more frequently (Table 4). As shown in Table 5, the overall in-hospital mortality was 23%. The survival rate of women was lower than men with type A dissection (*P* < 0.01). The surgical and nonsurgical outcomes were less favorable for women. In particular, type A dissection in women was associated with increased surgical mortality (*P* = 0.04) and nonsurgical mortality (*P* < 0.01).

**TABLE 3:**
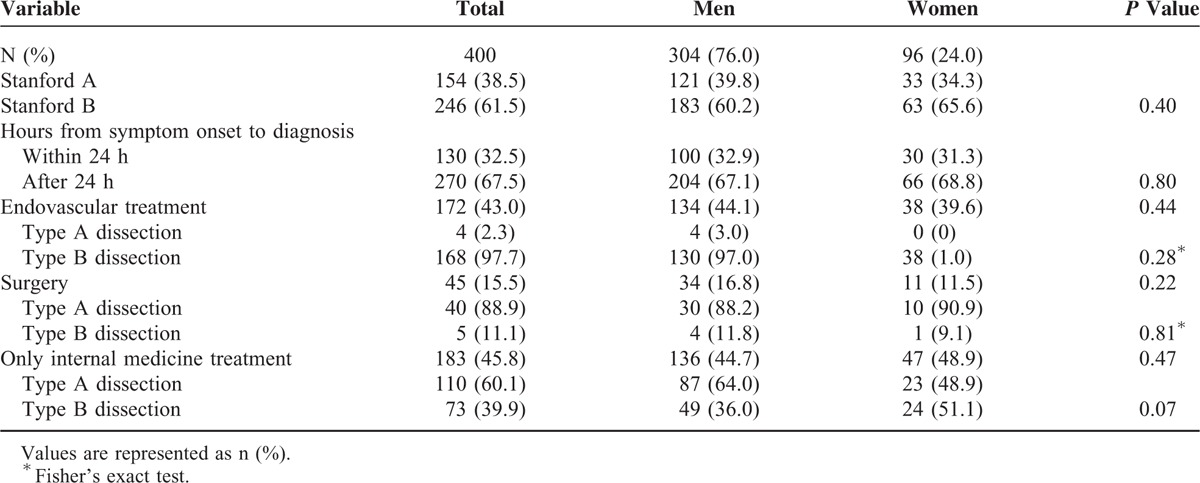
In-Hospital Treatment of Women and Men

**TABLE 4:**
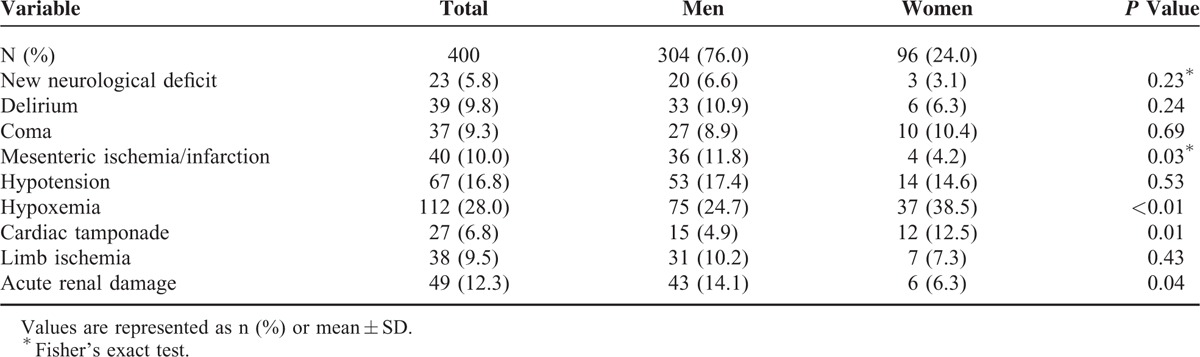
In-Hospital Complications of Women and Men

**TABLE 5:**
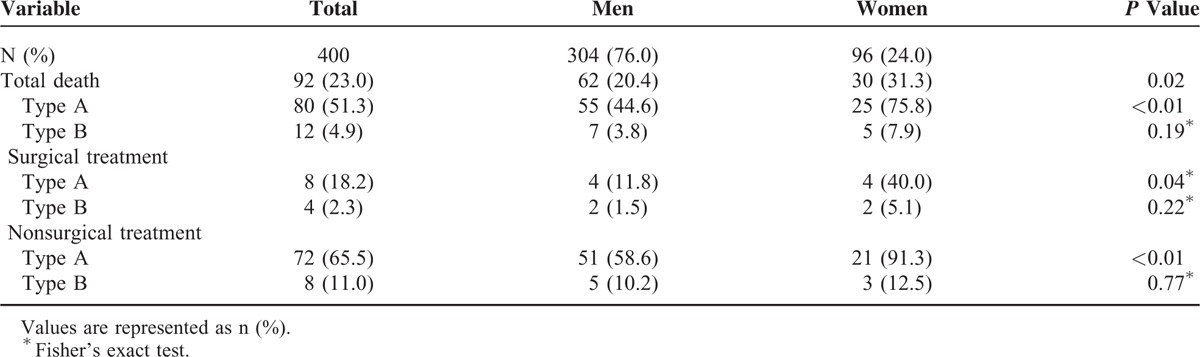
In-Hospital Mortality of Women and Men

## DISCUSSION

With the recognition of cardiovascular disease as the leading cause of global mortality among women, researchers focused on sex-related differences in AAD.^19–20^ The International Registry of Acute Aortic Dissection (IRAD) study^6^ evaluated differences in clinical features, outcomes of male and female with AD. The IRAD study reports that the ages of onset are 60.3 ± 13.7 and 66.7 ± 13.9 years for men and women, respectively. In contrast, in this study, we showed that the mean age of onset for men was earlier compared with women (49.6 ± 12.6 years and 54.2 ± 12.4 years, respectively), indicating that in the Xin Jiang region of China, the onset age of AAD is earlier than that in western countries. This difference related to a lower standard of healthcare, and unbalanced distribution of medical conditions between different regions in China.

In the present study, patients’ medical histories show that the number of men who smoked was significantly higher than that of women. Heavy smoking increases oxidative stress, induces endothelial injury, and eventually leads to expansion of aortic aneurysms.^21^

Pain is the most frequently reported presenting symptom of patients with AAD, regardless of age, sex, or other associated clinical complaints.^22–23^ Analysis of pooled data of >1000 patients in 8 studies showed that the pain of acute dissection is perceived as abrupt in onset and severe in 84% and 90% of patients, respectively.^24^ In our study, 79.75% patients presented with the classic onset symptoms of chest or back pain. Women more frequently described this pain as abrupt tearing or ripping, and men more frequently described the pain as sharp or stabbing. Migrating pain was described by 30% of patients, and 21.75% of patients presented with abdominal pain in the absence of chest pain, or with only abdominal distension (12.3%) accompanied by other uncommon symptoms such as hematemesis (1.8%), hematochezia (1.3%), coma/altered consciousness (2.8%), and delirium (2.8%). Dysphoria (17.1%), focal neurological deficits (17.8%), and pulse deficits (8.9%) were present more frequently in men. The cause of most episodes of pain was obstruction by the dissection flap, which can either prolapse across a vessel's origin without entering it or directly extend into a vessel.^25,26^

Imaging studies showed that pleural effusion, which was the most frequent (16.3%) pulmonary complication of AAD, was more frequent in men. Small effusions result from a nonhemorrhagic exudate, likely caused by inflammation. Large effusions mostly caused by blood leaking from the aorta into the pleural space.^27,28^ Other pulmonary complications of AAD include dissection-related atelectasis of pulmonary tissue and hypoxemia that were observed more frequently in women and may present with dyspnea as a prominent symptom.^29,30^

The present data show that mesenteric ischemia and acute renal damage were more frequent in men (Table 4). These results are consistent with the findings of computed tomography angiography (CTA), which showed involvement of the coeliac trunk and superior mesenteric arteries more frequently in men. Mesenteric ischemia is the most frequent gastrointestinal complication of AAD. However, by the time values of serum markers of bowel ischemia or infarction become positive, it is often too late to salvage the bowel or the patient. Therefore, it is essential to improve vigilance for mesenteric ischemia in every AAD patient with abdominal pain. Renal complications of AAD may be acute, or chronic.^31^ But physical examination was insensitive to renal ischemia early in the course of AAD.^32^

Heart is the most commonly implicated organ in progression of AAD. The heart-related complications mostly originated by disruption of dissection or aorta.^2^ For AAD, cardiac ischemia/infarction is an uncommon but critical complication.^2,33^ In our study, the frequencies of patients with myocardial ischemia and infarction were 8.3% and 4.3%, respectively, and women were more frequently affected by cardiac complications than men. Although the cardiac malperfusion combined with ECG changes in AAD patients, it is hard to distinguish from that of primary cardiac ischemia and infarction, which increase the possibility of misdiagnosis and improper treatment.^34,35^

Moreover, CTA and echocardiography results showed that pericardial effusion (34%) and tamponade (12.5%) were more frequent in women. Transudation of fluid across the thin wall of an adjacent false lumen into the pericardial space frequently causes pericardial effusion.^36^ Cardiac tamponade was diagnosed in 6.7% of our patients, mostly those presenting with acute Type A dissection, which is an ominous clinical predictor of poor outcomes as well as the leading cause of mortality in these patients.^37^

Among 1076 AAD patients in the IRAD study, nearly 50% were diagnosed and received treatment within 24 hours. In contrast, only 32% of patients were diagnosed within 24 hours in our study. Delays in diagnosis directly affect the prognosis of AAD patients.

The overall outcome of women was worse than that of men, and in particular, women with type A dissection had a higher death rate, including 30% higher in-hospital mortality than men. These results may explained, in part, for the reasons as follows: AAD occurs in women an average of 10 to 14 years later than men, advanced age is a risk factor for increased early or late death after surgery.^38,39^ Diagnostic imaging findings suggest that impending rupture occurs more often in women. In-hospital complications such as a higher incidence of myocardial ischemia/infarction, hypoxemia, and cardiac tamponade occur more frequently among women that characterizes their critically worse preoperative condition at an older age than that of men. In addition, most surgeons recommend immediate surgical repair for AAD patients.^40,41^ However, we found that women refused emergency surgery more often than men. There was no difference in the proportion of patients with type A or B dissection in the present study, and we found no significant variation between sexes in surgical technique, delay of surgery, or hemodynamics at surgery. The differences in postsurgical outcomes of women appear real, considering the possible association with advanced age and more frequent surgeries performed on patients at high risk.

## CONCLUSION

In the present study, we highlighted important differences in clinical characteristics, management, and outcomes between women and men with AAD. The average onset age of women was older than that of men; women had severer in-hospital complications, and higher in-hospital mortality. Therefore, physicians should heighten their suspicion of acute aortic dissection in women and its associated morbidity and mortality. The improvements in regional healthcare systems, early diagnosis, stringent control of hypertension, lipid profile optimization, smoking cessation, and other measures that reduce the risk of AAD. Further, the development of strategies to identify and treat female patients at high-risk may improve their clinical outcomes and reduce the overall mortality of AAD.

## LIMITATIONS

This study has some limitations. First, classification of patients as chronic or acute was not random, leading to a relatively small patient-sample size in the acute group. Further, its retrospective nature and small sample size account for the study's insufficient statistical power. Second, we used the Stanford, but not the Debakey classification method, the differences in clinical outcomes between Debakey Types I and II were not analyzed. Third, we lacked long-term follow-up of patients after discharge, and therefore, the prognosis of patients was limited to the duration of their hospitalization.
